# Compounding, not adding: a layered framework for why maternal health programs gain coverage without saving lives

**DOI:** 10.3389/fgwh.2026.1908098

**Published:** 2026-08-18

**Authors:** Shyamkumar Sriram

**Affiliations:** Department of Rehabilitation and Health Services, College of Public Affairs and Health Sciences, University of North Texas, Denton, TX, United States

**Keywords:** health equity, health systems framework, low- and middle-income countries, maternal mortality, social determinants of health, Three Delays Model, universal health coverage

## Abstract

For 30 years, investment in maternal health has delivered rising coverage: more antenatal visits, more skilled attendance, and more births inside facilities. However, survival has not kept pace. Each of the two frameworks that dominate the field explains one half of this issue. The Three Delays Model identifies where along the path to care a woman dies; the social determinants tradition explains why those delays fall where they do. There is no framework that connects the two. This Perspective sets out a framework that does, which I call the Compounding Vulnerability Framework. It is conceptualized as a pyramid: a broad base of structural root causes (poverty, gender inequality, schooling, and geography), a band of social and economic barriers above it, and the three care-seeking delays stacked higher still, narrowing to the apex where a preventable death occurs. The organizing idea is that these disadvantages multiply rather than add. A program that works on one layer alone—particularly the middle and upper layers that throw off visible coverage figures—will raise those figures and leave mortality almost unchanged, because the obstacle simply moves to the next layer left untouched. Read against the documented record of national programs, the evidence is consistent with this expectation. The framework turns the familiar argument about what works into a question about how many layers an investment reaches at once.

## Introduction

In 2023, approximately 260,000 women died during pregnancy or soon after childbirth—one death about every 2 min. Most of these deaths could have been prevented with clinical knowledge that has been available for more than a generation (1). What ought to trouble us about the past three decades is not any lack of progress. Much has moved: Antenatal attendance, skilled birth attendance, and facility delivery have all risen steeply across high-burden countries. Yet, national maternal mortality ratios have often declined far more slowly than those curves promised. The gap between how much care women now receive and how many of them live is the puzzle this Perspective addresses.

Two ideas provide most of the explanation in the field, and each, taken alone, accounts for only part of the gap. The Three Delays Model places a maternal death at one of three moments: delay in deciding to seek care, delay in getting to a facility, and delay in receiving adequate care once there (2). As an account of the final hours, it has no rival. The social determinants tradition works at the other end of the timescale, tracing how poverty, gender inequality, education or the lack of it, and geographical distance shape risk long before any complication begins (3, 4). What has been missing is an idea to hold the two ends together: a single structure showing how the conditions at the bottom feed into the delays at the top, in a form that one can use to read a program, or build one, rather than only to explain a death once it has happened.

That structure is what I propose in this Perspective. I have called it the Compounding Vulnerability Framework, and its claim is plain enough to state: The disadvantages bearing on a pregnant woman do not stand side by side and add up; they fold into one another and multiply. If this is correct, it explains the coverage-without-survival pattern more cleanly than either parent framework manages on its own. Mend one layer of the system and leave the rest standing, and the coverage number attached to that layer will rise, while the death rate barely stirs, because the binding constraint has moved up to the next layer that was untouched. By binding constraint, I mean the single layer whose repair would, at that moment, do most to raise survival, the point at which the path to a safe birth is actually severed; the term is borrowed from growth diagnostics in development economics, where relieving one issue moves the problem to the next-weakest layer rather than dissolving it (18). Stated as a rule for designing programs rather than as a postmortem, the framework suggests which investments should shift the survival curves and which should not. National program records can be read against this claim; the following cases illustrate the framework more than they test it, since they were selected for being instructive rather than sampled at random.

## The compounding vulnerability framework

**Figure 1:**
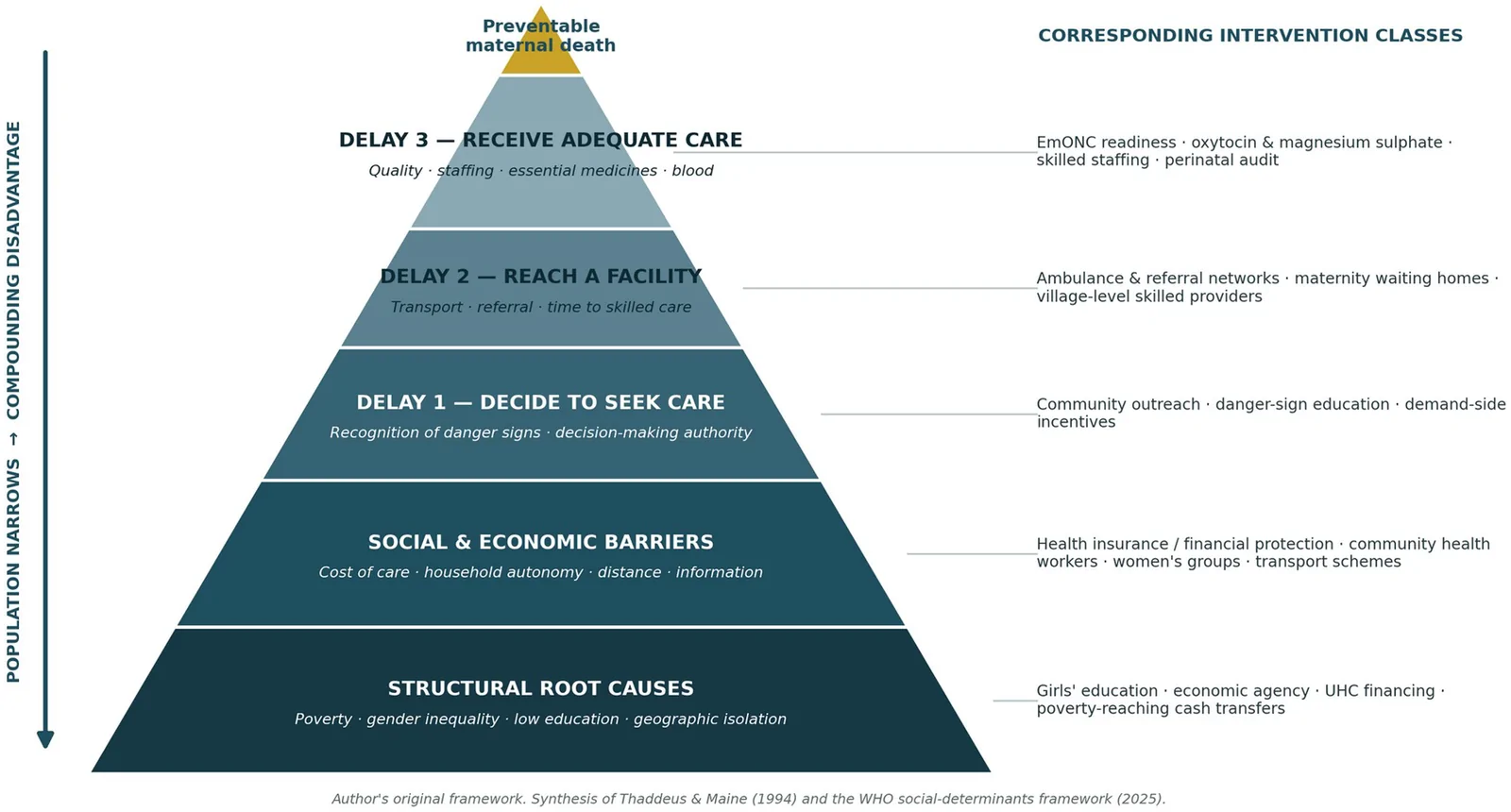
The Compounding Vulnerability Framework for maternal mortality. Five conditions are arranged from a wide structural base to a narrow apex of preventable death; the population at risk thins with height as disadvantage compounds. Each layer is paired with the class of evidence-based intervention known to work on it. The author's original framework, connecting the Three Delays Model (2) to the WHO social determinants framework (3).

Five conditions are stacked as a pyramid (Figure 1). The wide base represents the structural root causes: poverty, gender inequality, limited schooling for girls, and isolation by distance or terrain. Resting on this base is the band of social and economic barriers through which those root causes reach a particular pregnancy: what care costs, how much say a woman has in her own household, how far she lives from help, and what she knows. The top three tiers are the Three Delays set upright: deciding to go, getting there, and being treated properly on arrival. The apex, the narrowest point, is the outcome the whole structure exists to explain: a preventable maternal death.

The pyramid’s shape is significant. The base is widest because root causes touch almost everyone; in a high-burden setting, it is difficult to find a woman untouched by them. As one climbs the pyramid, the population narrows. Not every woman constrained by poverty also faces transport failure when labor turns dangerous; not every woman who reaches a clinic finds it without a midwife or without blood. The few who meet a wall at every level—the women at the apex—are the ones who die. What the pyramid makes visible, and what national averages hide, is that maternal death is not scattered at random across a population. It clusters, with grim predictability, wherever disadvantages stack up. A woman who is poor, unschooled, remote, and then sent to an underequipped facility is not carrying four separate risks. She is living one compounded risk, in which each hardship makes every other one heavier.

It helps to picture the arithmetic the metaphor implies. A woman's chance of survival can be thought of as the product of her odds at each layer: the odds of deciding in time, of reaching care, and of receiving adequate treatment on arrival—each a fraction somewhere between 0 and 1. These fractions multiply, not add. Raising one fraction from a half to near-certainty barely moves the product if another remains close to zero. This is exactly why a program can clear a whole layer and scarcely reduce the death rate. Addition would forgive a single weak link, letting strength in four places make up for collapse in the fifth; multiplication will not, because a near zero anywhere in the chain drags the whole product down with it. This is the formal content of compounding, and it is what separates the framework from a checklist of risks to be totted up and weighed. In its strongest form, this is a weakest-link relation: A near zero at any single layer caps the whole product regardless of the others, which is also why the reach score used later counts layers touched only as a rough proxy and not as the survival model itself.

Where does this framework go beyond its sources? It extends in two places. First, the construction is vertical rather than additive. The social-determinants literature furnishes the lower two layers and the Three Delays Model the upper three, but the seam between them is the contribution: the claim that the lower layers manufacture the upper ones. A woman's delay in deciding to seek care is not some free-standing quirk of behavior. It is, very often, what gender inequality and the price of care look like at the moment a complication starts. Each layer is then matched, in the right-hand column of Figure 1, with the class of intervention known to work on it. This turns a diagnosis into something nearer a design language. To read a program through the framework, one only asks two questions of it: which layers does it touch and how many.

This framework is not a relabeling of intersectionality or of the structural-determinants synthesis (5). Those bodies of literature have already established, past reasonable argument, that maternal risk is socially and structurally patterned. Crear-Perry and colleagues trace that patterning directly to the proximate mechanisms of maternal risk, so the framework does not reassert it. What it adds are three operational moves the synthesis does not make: It locates where along the care pathway patterned risk becomes fatal, pairs each layer with the intervention class known to act on it, and predicts where the binding constraint travels when one layer is repaired alone. What the framework adds is direction and use. For any given program, it indicates where the coverage gains should accumulate and where mortality will remain unchanged, placing the binding constraint at the lowest layer left untouched by the program, rather than at the layer the program happens to count.

This framework is not a restatement of the effective-coverage cascade, the measurement tradition that insists that access and survival are different things because the quality of care sits between them and that a high-quality health system—not mere contact with one—is what converts coverage into lives saved (6, 7). That work is indispensable, and the framework here leans on it without apology. The multiplicative arithmetic set out previously is shared with the cascade rather than being original to this framework; what differs is the question asked. The cascade follows a single service, asking how much of a given contact survives the passage from attendance to readiness to competent treatment to a measurable health gain. The Compounding Vulnerability Framework asks the prior question the cascade sets aside: Why is it that the woman who most needs a service is so often the same one obstructed at every layer beneath it, and where does the binding constraint travel when a program mends one layer but leaves the others standing? The cascade measures the leak in a single pipe; the framework maps which pipes a society has never laid, and predicts in advance where the next blockage will surface once the first is cleared. The two are complementary and not contradictory, and a program that scores well on one can still fail the other. A worked case makes the difference concrete. Take the MomConnect program through each lens. The cascade, applied to the service itself, asks how many enrolled women receive, understand, and act on each message, and how much health gain survives that chain; it might reasonably score the service a success. The framework asks instead where the binding constraint on survival now sits, given that MomConnect reaches only the deciding layer and, in part, the accountability side of the adequacy layer. It answers, at the transport layer and the structural base. It predicts that a well-run single-layer service will post high within-service coverage alongside a flat death rate. That cross-layer prediction is what the cascade, following one service at a time, does not itself yield.

## A testable prediction

The prediction can be stated in one line: Money spent on a single layer—particularly on the middle and upper layers that throw off visible coverage statistics—will buy access without buying survival, because the lower layers continue feeding women into a pathway the program has only half mended. The other side of the claim is that programs reaching the structural base, or several layers together, should deliver survival gains that single-layer programs rarely manage. What would overturn the framework is just as clear: a program that reaches the base and several layers with it, yet leaves mortality stalled exactly where a single-layer rival left it, or a narrowly single-layer program that drives survival down as steeply as the multilayer cases do. Either pattern, observed more than once and not explained away by measurement, would break the framework rather than bend it; a viewpoint that can survive any result is not worth proposing.

**Figure 2:**
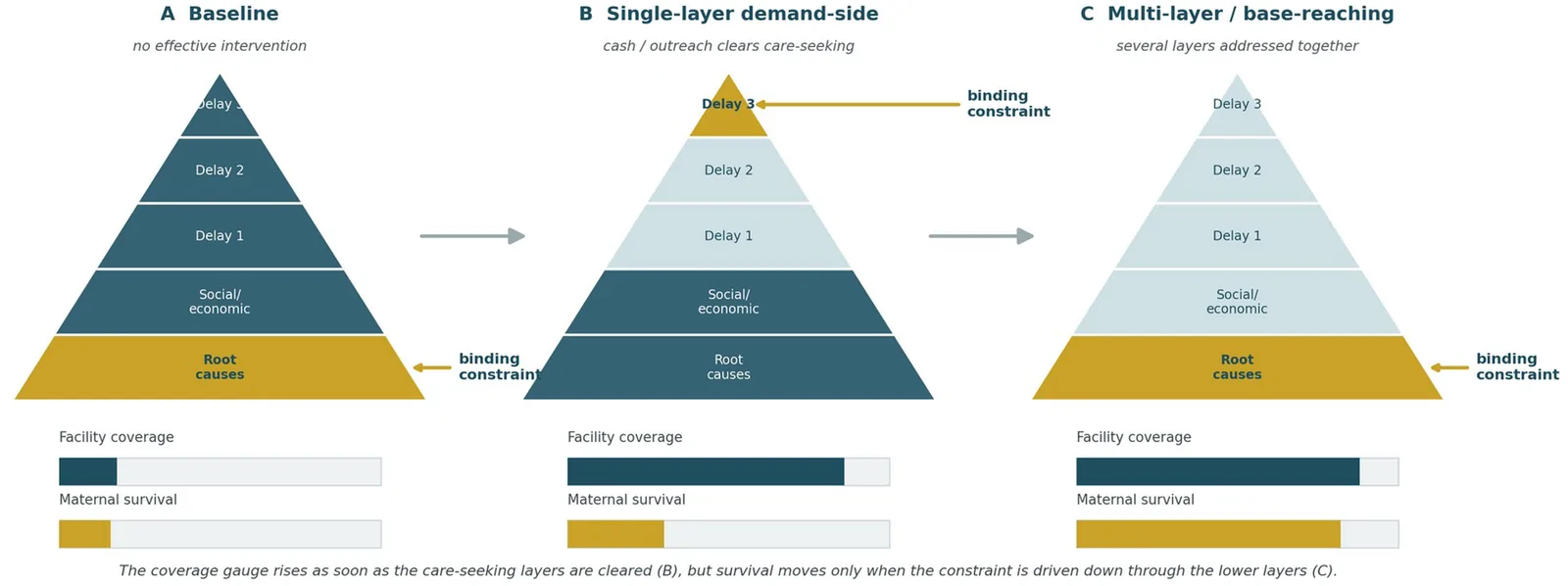
Migration of the binding constraint. **(A)** With no effective intervention, the constraint sits at the base and both coverage and survival are low. **(B)** A single-layer demand-side program clears the care-seeking layers; facility coverage rises sharply, but the constraint moves up to the adequacy-of-care layer and survival barely shifts. **(C)** A program reaching several layers, including the base, drives the constraint down and survival follows. The coverage and survival gauges diverge precisely in scenario B, which is the coverage-without-survival pattern the framework predicts.

The mechanism is migration (Figure 2). A demand-side push that succeeds in getting women to facilities does not remove the obstacle to survival; it relocates it—from the village to the labor ward, where a delivery room lacking the staff, the drugs, or the blood to manage a hemorrhage becomes the thing that kills. Coverage indicators climb, because they measure the layer that was fixed. Mortality indicators remain flat, because they answer to the layer left untouched. A program can therefore be a real success on its own terms yet a disappointment against the outcome that finally matters. Any evaluation resting on a single indicator will read it wrongly. Table 1 illustrates how five well-documented national programs map onto the five layers, scored by how firmly each reaches them. This mapping is illustrative, coded with knowledge of each program's outcome, and therefore open to the circularity noted later.

**Table 1:** Layer reach of five national maternal health programs.

Program	Structural root causes	Social and economic barriers	Delay 1: decide	Delay 2: reach	Delay 3: adequate care
Ethiopia: Health Extension Program	Absent	Partial	Strong	Strong	Partial
Rwanda: CHW + Mutuelles + PBF	Partial	Strong	Strong	Strong	Strong
Sri Lanka: universal free maternity care	Strong	Strong	Strong	Strong	Strong
India: Janani Suraksha Yojana	Partial	Partial	Strong	Partial	Weak
South Africa: MomConnect	Absent	Absent	Strong	Absent	Partial

Cells code the strength of each program's reach into each framework layer (Strong, Partial, Weak, or Absent). Programs whose components reach several layers (Rwanda, Sri Lanka) recorded the largest survival gains; programs acting forcefully on a single layer (India, South Africa) show the predicted coverage-without-survival ceiling. Coding is the author's analytic judgment, drawn from the cited program evaluations (8–14), and not a measured effect size. India's reach reflects the Janani Suraksha Yojana operating within the National Health Mission, which extends partial supply-side reach beyond the cash transfer alone.

## Evidence from national programs

Beginning with the case least exposed to the way these examples were chosen, in Brazil, Bolsa Familia—a poverty-reaching cash transfer program that was not selected here on any maternal outcome—has been associated with lower maternal mortality across the whole population (15). The lesson lies in naming the layer. Bolsa Familia works on the structural base across the entire life of a household, whereas a transfer pinned narrowly to attending a facility works only on the deciding layer—same instrument, different layer, different survival result, which is exactly what the framework leads one to expect. Because this association is independently reported and the case was not handpicked to fit, it anchors the argument on firmer ground than the program comparisons that follow.

Consider two programs whose parts spread across the pyramid instead of clustering on one band. Rwanda assembled three mechanisms, each working on more than one layer at once. An elected, specialized cadre of community health workers addressed the deciding and the reaching layers. Community-based insurance through the Mutuelles de Santé program lifted the fear of a catastrophic bill at the social and economic layer and was associated with markedly higher skilled attendance, with the largest gains being among the poorest women (8). Performance-based financing tied a facility' payment to a verified quality score rather than to sheer volume, directly targeting the adequacy of care (9). Because these overlapped, Rwanda posted one of the fastest sustained declines in maternal mortality on record. The plateau it reached after 2015 fits the framework rather than contradicting it: A program that has done its work at the base, middle, and top layers runs out of easy gains sooner or later and is left facing in-facility quality as the last barrier standing (10). That reach, it should be said, drew on administrative capacity unusual for Rwanda's income, so it is less a template than a demonstration. Sri Lanka illustrates the same logic played out over 70 years. Free institutional delivery has been in place since 1952, supported by a public health midwife answerable for every pregnant woman in her area, and continuous investment in district-level emergency obstetric care. Together, these interventions touch every layer of the pyramid, including the structural base, and have produced one of the most complete survival transitions achieved anywhere from a modest-income country (11). Seven decades is the point as much as the layers: The transition presupposes sustained investment that few ministries can promise, which is the cost the “reach more layers” rule quietly implies.

Now, for the single-layer cases, where the ceiling shows itself, India's Janani Suraksha Yojana, the largest conditional cash transfer for safe motherhood in the world, lifted facility delivery from roughly 39% to 89% in under two decades. Over the same span of time, the maternal mortality ratio fell substantially, from approximately 254 per 100,000 live births in 2004–2006 to 88 in 2021–2023, a reduction of nearly 65% (17). The framework does not read this as failure but as a coverage–survival gap of a particular shape. Coverage approached saturation, while the ratio settled at 88, still above the SDG threshold of 70 and roughly three times Sri Lanka's, which places the last barrier to survival above the layer the cash transfer paid for. The residual deaths are concentrated at the adequacy-of-care layer—obstetric hemorrhage with delayed diagnosis and weak interfacility referral—the layer a demand-side transfer does not repair (12, 13). The survival gain India did achieve should not be attributed to the cash transfer alone: The program operated inside the National Health Mission, which acted at the same time on supply-side layers through infrastructure, workforce, and free-entitlement packages, against two decades of secular growth. India was therefore never a pure single-layer case, and its partial success is what multilayer reach predicts. The sharpest evidence is internal: Deaths cluster in poorer states where the base and adequacy layers stay weak, and are dispersed where those layers are strong—the compounding pattern inside one national border. South Africa's MomConnect teaches the same lesson from the digital side. As a national mobile health service, it works on the deciding layer by putting stage-specific guidance and danger-sign information into women's hands, and it opens an accountability line at the adequacy layer through its feedback helpdesk. It does nothing for transport and nothing for the structural base, nor was it meant to (14). It is an upper-and-partial-middle instrument resting on a foundation it does not try to repair, and its reach ends accordingly.

One case the selection here cannot have manufactured is a high-income one. In the United Kingdom, universal, free, and high-quality clinical access has, in effect, repaired the upper layers. However, confidential enquiries still find maternal deaths clustering by deprivation and by ethnicity, that is, at the structural base (16). A strong apex has not lifted the ceiling, because the base was never leveled. This is the framework's prediction observed where access is not the binding constraint and where the case was not chosen to fit, which is why it carries more weight than any single purposively selected program.

## Implications for policy and measurement

What should change if this framework is correct? Three things are needed, mostly about where we choose to look. A coverage figure cannot stand alone as the measure of a program: a rising facility delivery rate sits comfortably beside a flat death rate whenever the adequacy-of-care layer lags behind the care-seeking layers below it. Indicators of quality and structural base—medicines on the shelf, a skilled attendant present at birth, emergency obstetric readiness, a completed perinatal audit, girls in school, and families shielded from ruinous bills—deserve the same scrutiny as access numbers. Programs, in turn, are better judged by how many layers they reach than by the one indicator they were built to move. The useful question is rarely whether a community worker scheme beats an insurance scheme in the abstract, but how many layers a given combination covers between them. The long-running quarrel over what works begins to look like what it usually is—a disagreement between champions of different single layers—once the framework recasts it as a question of architecture, in which the number of layers an investment links matters more than the merits of any single one.

A caveat on use should be stated. The framework identifies where the binding constraint sits; it does not, by itself, tell a budget-constrained ministry which layer to fund first. Two things temper the reach-more-layers implication. The first is cost and capacity: Base and adequacy-of-care work is slow, expensive, and administratively demanding, and the admired transitions rested on decades of investment or unusual state capacity, so neither transfers wholesale. The second is political economy: Demand-side instruments persist because they are cheap, fast, simple to run, and yield a coverage figure that is measurable and politically attributable within an electoral cycle. The framework's role here is diagnostic rather than prescriptive; it names the layer that will constrain survival next, so a sequenced plan can fund the lowest binding layer it can reach rather than the most visible one. Which layer returns the most survival per unit spent, given what is already in place, is the optimization the framework frames but does not solve.

**Figure 3:**
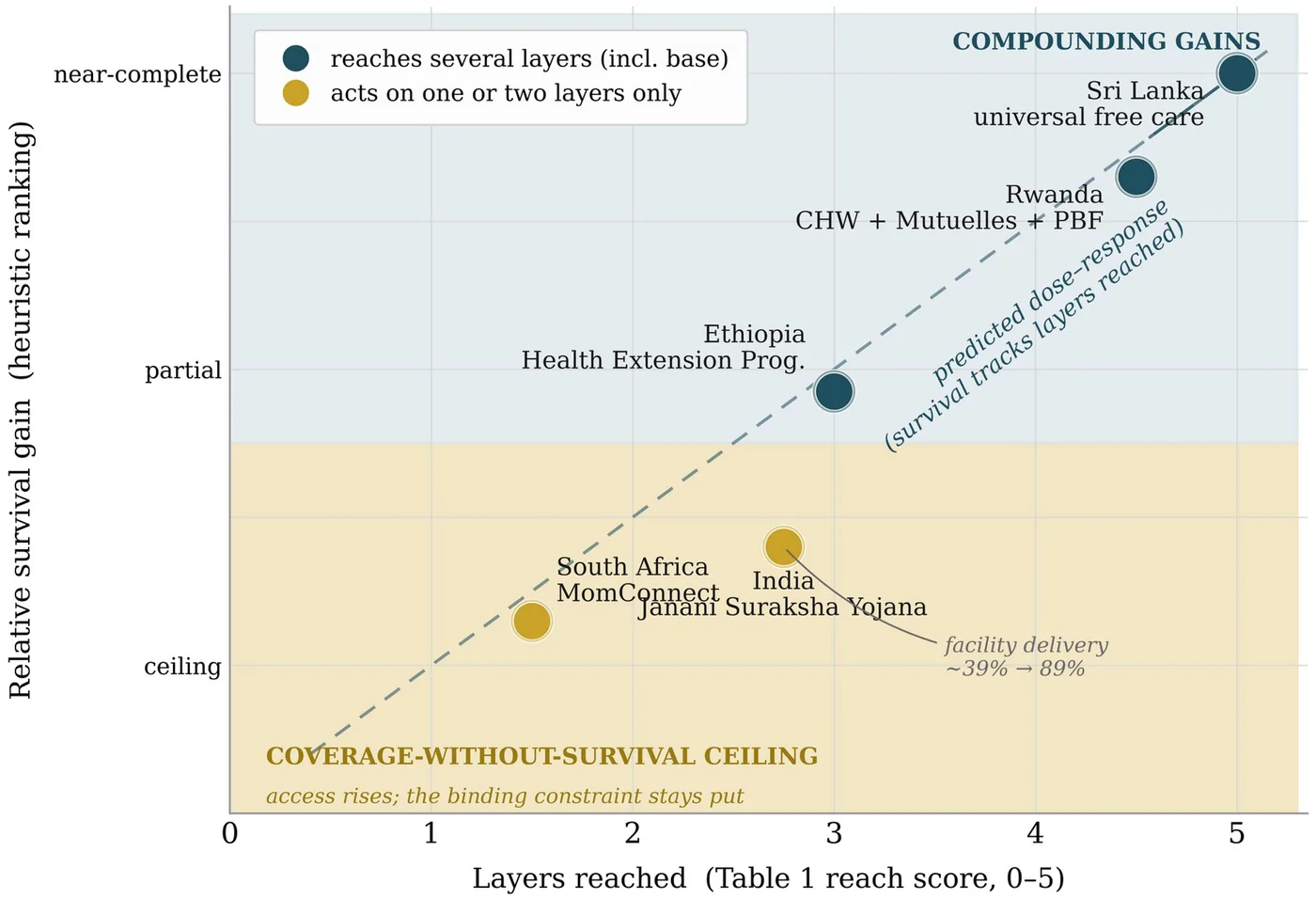
Layers reached against survival gain for the five programs in Table 1. The horizontal axis is each program's reach score, formed by summing its layer codes from Table 1 (Strong = 1, Partial = 0.5, Weak = 0.25, Absent = 0) across the five layers; the vertical axis is a heuristic ranking of survival gain, the author's ordinal ranking read from the reported mortality trajectories, encoding order only and assigned with knowledge of outcomes. Programs acting on one or two layers (India, South Africa) settle in the lower band, where coverage can climb, while survival stalls; programs reaching several layers, the structural base included (Ethiopia, Rwanda, Sri Lanka), sit higher, in the direction the framework expects. The annotated jump in India's facility delivery rate (roughly 39%–89%) marks the coverage-without-survival gap directly. Positions encode the author's analytic judgment and not effect estimates, and the figure is offered as an illustration and not as proof.

None of this needs to remain abstract. The reach categories in Table 1 could be hardened into a simple scored instrument, each program rated layer by layer and the resulting profile set against its mortality trajectory, as Figure 3 does for the five programs scored here. A funder or a ministry could then see, before committing, which layers a proposed portfolio leaves bare, and the framework would graduate from a way of reading programs into a way of planning them.

## Limitations

A word on what the framework is not. It is a heuristic and not a fitted causal model. Scoring how far a program reaches into each layer is a matter of judgment, and careful readers will dispute the margins. The strength categories in Table 1 are reasoned assignments and not measured effect sizes. The reach score used in Figure 3 sums those categories only as a rough count of layers touched; it is a proxy for reach and not the multiplicative survival model the framework proposes. The cases mentioned here were chosen because they succeeded or failed in instructive ways, which is no basis for strong causal inference. There is a circularity to guard against, too: Layers scored in full knowledge of how each program fared can quietly read the outcome back into the coding. The present Table 1 makes no claim to have escaped this, and is offered as an illustration and not as proof. The obvious next step is to build the scored instrument described herein and test it prospectively, against outcome trajectories, across a larger and less handpicked set of programs, with the layer scoring fixed before the mortality record is consulted. In this way, the framework would be made to predict rather than merely recognize what it has already seen. As set out earlier, this is the concrete form of the falsifiability criterion: A base-reaching program that leaves mortality where a single-layer rival left it—or a single-layer program that drives survival down as steeply as the multilayer cases—would break the framework rather than bend it.

## Conclusion

Maternal death is not a riddle awaiting discovery. It is a structural failure with a knowable shape, in which disadvantage at the base of a society accumulates, layer upon layer, into the deaths crowded at the top. The Compounding Vulnerability Framework is an attempt to make that accumulation visible and, having made it visible, to make it useful—to anticipate where coverage will rise without survival and where it will not. To name the real cause of a woman's death, and the layer at which it could have been stopped, is not a rhetorical flourish. It is the first step for any response that intends to work.

## Data availability statement

This Perspective draws entirely on published sources, all of which are cited in the article. No new data were generated or analyzed. Further inquiries can be directed to the corresponding author.
